# Dynamic versus Adynamic Graciloplasty in Treatment of End-Stage Fecal Incontinence: Is the Implantation of the Pacemaker Really Necessary? 12-Month Follow-Up in a Clinical, Physiological, and Functional Study

**DOI:** 10.1155/2015/698516

**Published:** 2015-03-11

**Authors:** Piotr Walega, Michal Romaniszyn, Benita Siarkiewicz, Dorota Zelazny

**Affiliations:** ^1^Third Department of General Surgery, Jagiellonian University Medical College, Pradnicka Street 35-37, 31-202 Krakow, Poland; ^2^Gabriel Narutowicz Memorial Hospital, Pradnicka Street 35-37, 31-202 Krakow, Poland

## Abstract

*Purpose*. The aim of the study is to compare functional results of end-stage fecal incontinence treatment with dynamic graciloplasty and adynamic graciloplasty augmented with transanal conditioning of the transposed muscle. *Methods*. A total of 20 patients were qualified for graciloplasty procedure due to end-stage fecal incontinence. 7 patients underwent dynamic graciloplasty (DGP), whereas 13 patients were treated with adynamic graciloplasty, with transanal stimulation in the postoperative period (AGP). Clinical, functional, and quality of life assessments were performed 3, 6, and 12 months after the procedures. *Results*. There were no intraoperative or early postoperative complications. The detachment of gracilis muscle tendon was observed in one patient in DGP group and two in AGP group. There was a significant improvement of Fecal Incontinence Quality of Life (FIQL) and Fecal Incontinence Severity Index (FISI) scores in both groups 12 months after procedure. Anorectal manometry showed improvement regarding basal and squeeze pressures in both groups, with significantly better squeeze pressures in AGP group. *Conclusions*. The functional effects in the DGP and AGP groups were similar. Significantly lower price of the procedure and avoidance of implant-related complication risk suggest the attractiveness of the AGP method augmented by transanal stimulation.

## 1. Introduction

Approximately 7–10% of the working population suffers from fecal incontinence, about 30% of which is affected by the end-stage of the disease. This includes patients with congenital absence of the anal sphincter, patients after surgical treatment of the anus and rectum, and also after major perineal trauma, the spinal injury or damage of peripheral innervation of sphincters [1], who in most cases do not respond to conservative measures, such as biofeedback training or electrical stimulation, and are not eligible for surgical repair, such as sphincteroplasty. To improve quality of life and avoid the end-stoma, this group requires the “last chance methods”: sphincter reconstruction by procedures involving transposition of autologous muscle or replacing anal sphincter with artificial prosthesis [2]. Regarding muscle transposition procedures, most commonly used procedures are the unstimulated graciloplasty (AGP) and dynamic (stimulated) graciloplasty (DGP), which were proven successful in highly selected patients [3].

Despite the fact that dynamic graciloplasty became more popular than its unstimulated variant, Rosen et al. showed that after initial conditioning of the transposed muscle even half of the patients no longer benefit from electrostimulation a year after the treatment [4]. Moreover, a large percentage of patients reported constipation as adverse effect of the procedure, even when the stimulation was turned off [5]. The data coming from literature reviews showed that the risk of complications was similar in both procedures; however the most severe complications were correlated with infection of the stimulator site or electrodes; thus revision and stimulator explantation rate in DGP is high [6]. The question emerged whether the pacemaker implantation is necessary for the success of the operation. There was no sufficient data comparing dynamic graciloplasty and adynamic (unstimulated) graciloplasty, partially because these procedures are rarely carried out, only in highly specialized units. Therefore, we designed a clinical study to compare the results of patients' functionality and satisfaction after dynamic graciloplasty and adynamic graciloplasty.

## 2. Purpose

The aim of the study is to compare functional results of end-stage fecal incontinence treatment with dynamic graciloplasty and adynamic graciloplasty augmented with transanal conditioning of the transposed muscle.

## 3. Methods

This was a prospective observational study. In this study, based on the IFGD guidelines, the following stages of research were used

- identification and assessment of the epidemiological control of defecation based on subjective data, function tests, electrophysiological measurements, and imaging,
- the patient division into two groups:
  - patients treated by dynamic graciloplasty (DGP) and
  - patients treated by adynamic graciloplasty (AGP),
- analysis of complications during and after surgery and functional tests 3,6, and 12 months after surgery,
- assessment of the Fecal Incontinence Severity Index (FISI) [7] and Fecal Incontinence Quality of Life (FIQL) at 3, 6, and 12 months after surgery.

### 3.1. Inclusion Criteria

- End-stage fecal incontinence (>35 points in Fecal Incontinence Severity Index),
- lack of sphincter or extensive sphincter defect (more than 1/3 of circumference of the muscle based on a transanal ultrasound examination),
- unsuccessful previous treatments: electrostimulation (ES), biofeedback + ES, sphincteroplasty, and SNS,
- sphincter innervation defect (verified by electrophysiological examination).

### 3.2. Exclusion Criteria

- Age less than 18 and more than 70 years,
- contraindications to graciloplasty including patient's refusal to undergo the procedure, peripheral neuropathy (e.g., diabetic and polyneuropathic), ischemic limb disorders, severe heart, kidney, liver conditions, and so forth,
- contraindications to electrical stimulation usage.

A total of 20 patients were qualified for graciloplasty procedure during the period of 6 years, 14 women and 6 men; the mean age was 48 years (range 19–70 yrs). There were 8 Mediatronic implantable stimulators on the Investigators' disposal. Short expiry date of the devices and long qualification procedure forced the Investigators to withdraw initially planned randomisation, and to qualify first 8 consecutive patients to dynamic graciloplasty group instead. One of the stimulators was found defective prior to implantation, so there were a total of 7 patients in the DGP group, and 13 in AGP group. Both groups had similar gender and age characteristics (differences not significant).

### 3.3. Technique of Graciloplasty Procedure

**Figure 1:**
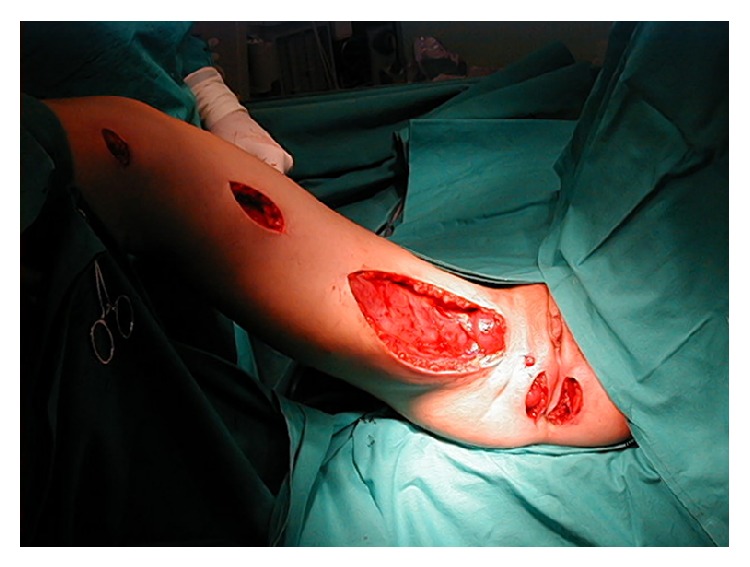
Graciloplasty procedure, wound configuration after gracilis muscle transposition.

In both groups of patients (DGP and AGP), the procedure was performed under general anesthesia. The gracilis muscle innervation was detected preoperatively by surface electromyography with a flexible array of 16 equally spaced silver bar electrodes and amplification system (ICU EMG16 Bioelettronica, Turin) as described by Bottin et al. [8]. Two 5 cm longitudinal incisions along the length of the thigh allowed the identification and mobilization of gracilis muscle. The classical method of operation described by Baeten et al. [9] or split-sling modification (Rosen et al.) [4] were performed. Gracilis muscle was dissected at the level of the neurovascular bundle (which was identificated intraoperatively by electrostimulator needle: Neuro-pulse Aaron Medical Industries, Inc.). Gracilis muscle was formed into the alpha or gamma loop, depending on the anatomical conditions and fitted around the rectum creating thereby a new sphincter muscle (Figure 1).

In the DGP group the next step was the attachment of two monopolar electrodes to the muscle (model 4300 Mediatronic) in the area of the main neurovascular bundle. Then the best parameters stimulation were defined. The most frequent parameters of the stimulation were: amplitude from 0.5 to 1.0 V, pulse width 0.210 ms, pulse frequency of 10 to 20 Hz (pacemaker IPG InterStim Mediatronic 3023). Electrostimulation was not used for the first week after surgery. The elastic bandage was maintained on the leg for 2-3 weeks. Drainage was removed from the wound 2-3 days after surgery. The patient was discharged home on average 5–7 days after surgery. In DGP group, two weeks after the procedure the prestimulation of muscle was started in order to achieve muscle fiber conversion (conditioning). Continuous stimulation was applied (voltage 1.5 V, pulse width 0,210 ms, frequency of 5.2 Hz). After 2 weeks, the stimulation was increased to 16 Hz (under stimulation parameters and anal canal pressures control) during the next 6 months. The output parameters of stimulation were maintained in case of 5 patients. In case of 1 patient the amplitude was increased to 2.0 V, and 1 patient required the electrodes polarity change, the increase of amplitude to 3.0 V, and frequency of stimulation. 3 patients with colostomy who had satisfactory functional results of treatment were qualified for stoma closure. It was performed in the period of 2-3 months after dynamic graciloplasty.

In AGP group the transanal stimulation by endoanal TNS stimulator SM1 (Schwa Medico) was implemented after healing of the wounds (about 2-3 weeks). The constant pulse frequency of 60 Hz were applied, 20–30 min three times a day.

### 3.4. Statistical Methods

Statistical analysis was performed by using the Mann-Whitney and Kruskal-Wallis test, which described the dependence of group-specific parameters, and which assumed the level of statistical significance *P* < 0.05. Friedmans' test was used to determine the temporal relation to the individual parameters in the studied groups. Calculations were performed using STATISTICA software.

## 4. Results

There were no intraoperative or early postoperative complications in our survey. The detachment of gracilis muscle tendon was observed in one patient in DGP group. This patient was scheduled for reoperation—due to evident atrophy of the transposed muscle, observed intraoperatively, the patient underwent a repeated graciloplasty using the other muscle. The electrostimulation was performed in 6 weeks after operation and it gave good functional results. Six months after the second treatment a gradually rapid muscular function atrophy was observed and confirmed by manometric examination. The electrophysiological assessment revealed a total lack of response to stimulation and ultrasound tests showed fast atrophy of gracilis muscle. Therefore, the patient was qualified to artificial anal sphincter implantation and excluded from further follow-up in this study.

One patient in AGP group suffered from the limb wound suppuration. The wound was treated conservatively and the antibiotic was administered with a satisfying result. Additionally, the same patient had symptoms of deep vein thrombosis in operated limb several months after surgery. It was treated conservatively. In case of two patients from AGP group the detachment of the tendon was observed and required reoperation without further complications.

### 4.1. Assessment of Defecation Control Score (FISI), and Quality of Life Score (FILQL)

**Figure 2:**
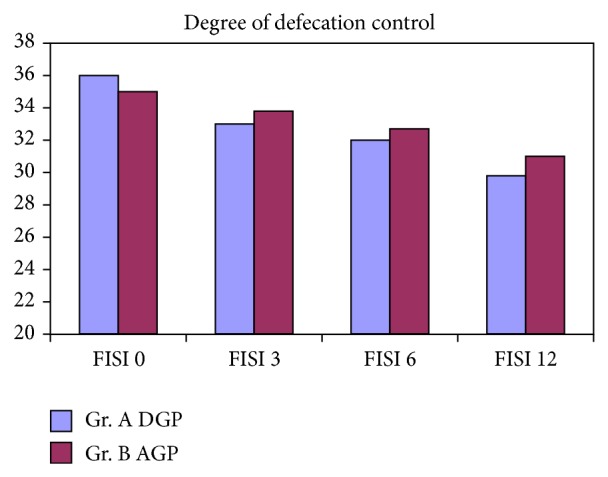
Fecal incontinence assessments.

Defecation control improvements were observed in both groups. In Jorge-Wexner questionnaire assessment, the patients scored from 11.5 points before surgery to 11.3 points in 6 months after surgery and 10.1 points in 12 months after surgery, the differences did not reach statistical significance (Friedman *P* = 0.07). However, the FISI score assessments ranged from 36,0 before to 29,8 points after treatment and were statistically significant (*P* < 0.05) (Figure 2). There was a significant (*P* < 0.05) improvement of Fecal Incontinence Quality of Life (FIQL) scores in both groups in 12 months after procedure. In DGP group patients felt embarrassed much more rarely (FIQL-E 1,8 before versus 3,3 points after) than in AGP group (1,8 versus 2,2 points) and the difference was significant (*P* < 0.05).

### 4.2. Functional Examination and Electrophysiological Evaluation

There was no significant difference in the initial basal pressure (BAP) and squeeze pressure (SAP) values between the groups (Mann-Whitney, *P* = 0.077). After the procedure, mean BAP and SAP values increased gradually and significantly in both groups (*P* < 0.05). There were no significant differences in BAP values between DGP and AGP group during the follow-up study. In AGP group, beginning from assessment 3 months after surgery, SAP values were significantly higher than in DGP group. This difference increased during the observation 6 and 12 months after surgery (Table 1).

In DGP group the pressure in the anal canal increased on average by 37 cm H_2_O above the basic pressure at the time of pacemaker activation. Interestingly, only 2 patients still used a stimulation permanently 6 months after operation. The remaining 4 patients activated the stimulator only occasionally. They claimed that it was not necessary for adequate control of defecation.

## 5. Discussion

During the last 50 years several European, American, and Australian centers used various modifications of muscle transposition or artificial implant in cases of patients with end-stage Fecal Incontinence. Belyaev and coworkers carried out a review of literature on transposition of the gracilis muscle with stimulator implantation and implantation of an artificial sphincter in 2006. In case of 378 patients (46.2%) the surgical revision had to be performed and in case of 259 patients (32.5%) the stimulator and electrodes had to be explanted. In as many as 82.8% of patients the postoperative complications were observed [2]. Other authors also report high incidence of complications of various degree [10, 11]. An analysis of literature carried out by Barisic and Krivokapic showed that adynamic graciloplasty is effective in approx. 50% of cases, and dynamic—from 45 to 80%, dependent on the experience of units performing the procedure (on average slightly above 60%). It is worth noting, that adynamic (unstimulated) procedures analyzed by the authors did not involve any conditioning of the muscle, therefore high failure rate might be due to muscle fatigue and the inability of patients to voluntarily contract the transposed fast-twitching muscle [12]. It has been shown previously by many authors, that one of the key factors determining proper function of the transposed muscle is muscle transformation from fast-twitching to slow-twitching fibers [13], however the method of conditioning not necessarily involves implantation of the stimulator.

Therefore, the question is whether it is necessary to perform implantation of costly electrostimulatory system (dynamic graciloplasty). A review of the literature prepared by Ruthmann et al. showed that removing the whole or part of the stimulation system due to implantation site infection or injury affected 6–42% of patients. Consequently, the high cost of the procedure becomes even higher [14].

In our study there was no need to remove the implant in any patient in the DGP group. A tissue atrophy of the transplanted gracilis muscle was observed in one patient despite performed stimulation. What's more, it turned out that in our study group, some DGP patients only temporarily needed to use electrostimulation when they suffered from loose stools. On the basis of the previous experience in treatment of fecal incontinence after low anterior resection syndrome by using electrical stimulation (ES), a transrectal stimulation of the transposed muscle was introduced in AGP group. As mentioned before, in our study the results achieved by transanal stimulation were even better than in the dynamic graciloplasty group. Such results can be explained by the fact that in AGP group the regular stimulation of the muscles affected not only the transposed gracilis muscle, but at the same time stimulated the residual sphincter and musculus levator ani, or other muscles of the pelvic floor [15]. Violi et al. who implanted stimulators selectively during the total reconstruction of perineum with both gracilis muscles also achieved better results in patients who underwent graciloplasty without implantation of the stimulator, and had their muscles stimulated by external device. Their conclusion was that muscle tone increase achieved by constant stimulation with an implanted device was sometimes not enough to provide satisfactory continence, whereas intermittent external stimulation enabled the patients to develop “pseudo-continence” yielding better bowel control [16]. Seccia et al. however achieved different results, with 71% success rate in externally stimulated graciloplasties, and 100% success rate in a small group who underwent dynamic graciloplasty [17].

One of our patients experienced an atrophy of the transposed muscle, which led to poor functional results. The effect of the gradual loss of muscle mass and strength was the reason for the failure of Yoshioka and Keighley treatment [18] and supposedly is responsible for some of the failures reported by other authors [19]. This may be either surgery related (damage of the neurovascular bundle intraoperatively), or patient-specific factor. In our case, since the patient experienced failure of the second gracilis transposition, we suspect the latter.

Most of the publications report good influence of the graciloplasty procedure on the patients' quality of life, provided that the neo-sphincter enables satisfactory continence, even with concomitant evacuatory problems [20].

Our observations lead to the conclusion that graciloplasty without pacemaker implantation could be an efficient and effective method of treatment of end-stage fecal incontinence as well. The condition for success in this type of treatment is muscle conditioning and exercise by means of transanal stimulation. The cost of a portable external electrostimulator is much lower (less than $300) than of the implantable device, it is safe and easy to use.

## 6. Conclusions

The presented results show that the rate of graciloplasty complications associated with pacemaker implantation can be potentially reduced by performing adynamic graciloplasty combined with simultaneous transanal stimulation. The functional effects of the DGP and AGP in presented survey were similar. Significantly lower price of the procedure and avoidance of implant-related complications risk, suggest the attractiveness of the AGP method augmented by transanal stimulation.

## Figures and Tables

**Table 1:** Manometric examination results (BAP: basal anal pressure, SAP: squeeze anal pressure).

	Before surgery		3-month follow-up		6-month follow-up		12-month follow-up	
	BAP (cmH_2_O)	SAP (cmH_2_O)	BAP (cmH_2_O)	SAP (cmH_2_O)	BAP (cmH_2_O)	SAP (cmH_2_O)	BAP (cmH_2_O)	SAP (cmH_2_O)
DGP	17 ± 11	35 ± 15	31 ± 12	55 ± 23	45 ± 19	57 ± 21	48 ± 19	51 ± 22
AGP	21 ± 15	39 ± 18	45 ± 21	82 ± 45	49 ± 19	116 ± 47	40 ± 15	108 ± 41
